# Yoghurt as a deglutition aid for oral medication: effects on famotidine powder dissolution rate and pharmacokinetics

**DOI:** 10.1038/s41598-023-29258-9

**Published:** 2023-02-03

**Authors:** Yayoi Gotoh, Yoshiyuki Tabata, Shinya Sugiura, Michiko Obara, Takashi Tomita, Toshio Suzuki

**Affiliations:** 1 Fujicco Co., Ltd., 6-13-4 Minatojima-Nakamachi, Chuo-Ku, Kobe, Hyogo 650-8558 Japan; 2 Sugi Pharmacy Co., Ltd., 62-1, Shin’e, Yokone-Machi, Obu, Aichi 474-0011 Japan; 3 Doctoral Program in Pharmaceutical Sciences, Graduate School of Pharmaceutical Sciences, Teikyo Heisei University, 4-21-2 Nakano, Nakano-Ku, Tokyo, 164-8530 Japan; 4 Department of Pharmaceutical Sciences, Faculty of Pharmaceutical Sciences, Teikyo Heisei University, 4-21-2 Nakano, Nakano-Ku, Tokyo, 164-8530 Japan

**Keywords:** Health care, Drug regulation, Geriatrics

## Abstract

Deglutition aid foods are used to help patients with dysphagia take oral medications. Yoghurt is occasionally used to help swallow medications; however, its influence on pharmacokinetics is poorly understood. Yoghurt made with *Lactococcus cremoris* subsp. *cremoris* FC has a characteristic viscous texture that facilitates bolus formation and deglutition due to its metabolite exopolysaccharide. We assessed yoghurt prepared with *L. cremoris* FC as a food deglutition aid. We performed a dissolution test using famotidine powder mixed with yoghurt and a food thickener. Famotidine dissolution rates without deglutition-assisting foods and with yoghurt or food thickener were 102.3 ± 1.7, 85.7 ± 4.6, and 46.4 ± 1.1% after 15 min, respectively. Next, we orally administered famotidine powder with water, yoghurt, and food thickener to rats and measured plasma famotidine levels. We observed no significant differences between all test groups. The T_max_ of famotidine mixed with a food thickener was significantly lower than that with yoghurt. These results suggest that yoghurt with *L. cremoris* FC did not remarkably affect the dissolution and pharmacokinetic profiles of famotidine powder. Thus, the administration of famotidine with yoghurt might be a suitable alternative to powder administration as a deglutition aid for patients.

## Introduction

Age-associated and sensory motor disorders, such as dysphagia, have increased in ageing societies. Patients with dysphagia have difficulty eating and swallowing food and are at risk of aspiration^1–3^. Therefore, food texture is modified by food thickeners, such as xanthan gum, for patients with dysphagia^4^. Similarly, owing to the difficulty of oral medication intake in these patients^5,6^, it is recommended to swallow medications with deglutition aid foods, such as food thickeners and jelly^7,8^. However, deglutition aids can affect drug disintegration and dissolution in vitro and the pharmacokinetics of medical ingredients in vivo^9–13^. Tomita et al.^12^ reported that food thickeners delayed the disintegration of mitiglinide tablets, rapid-acting insulin secretion-stimulating agents used to treat type two diabetes, and cancelled their suppressive effect on postprandial glucose absorption. Thus, it is important to verify the drug dissolution behaviour and pharmacokinetics when using deglutition aids.

Yoghurt is occasionally used as a deglutition aid for oral medication in patients with dysphagia in nursing care because of its soft and fluid texture^14,15^, and its texture depends on the type of lactic acid bacteria used for manufacturing. Some strains of lactic acid bacteria are known to improve the texture and viscosity of dairy foods by producing exopolysaccharides during fermentation^16^. *Lactococcus cremoris* subsp. *cremoris* FC (*L. cremoris* FC) is a lactic acid bacteria originally isolated from fermented milk in the Caucasus region^17^. Fermented milk with *L. cremoris* FC has a viscous texture owing to the production of exopolysaccharides. We have previously reported that yoghurt made with *L. cremoris* FC has characteristic physical properties that facilitate bolus formation and deglutition due to high cohesiveness; thus, patients with dysphagia are less likely to aspirate yoghurt fermented with *L. cremoris* FC rather than with *Lactobacillus bulgaricus* and *Streptococcus thermophiles*^18^. Matsuo et al.^19^ verified that yoghurt fermented with *L. cremoris* FC did not remarkably affect the disintegration or dissolution profiles of magnesium oxide tablets. Therefore, yoghurt may help patients with dysphagia to take oral medications. Magnesium oxide tablets are often prescribed to the elderly as a laxative, and their effect is occasionally suppressed by food thickener as a deglutition aid^15^. In the future, we plan on conducting a clinical study to verify the efficacy of magnesium oxide tablets with yoghurt. However, the effects of yoghurt on pharmacokinetics have not been revealed. In addition, magnesium oxide is not suitable for a pharmacokinetic study because of its low absorption into the blood.

In this study, we assessed yoghurt with *L. cremoris* FC as a deglutition aid. We used famotidine powder, which decreases stomach acid production and is used to treat acid-related gastrointestinal conditions. Since famotidine is available both by prescription and over-the-counter, it is widely available on the market. We investigated the dissolution rate of famotidine mixed with yoghurt in vitro and pharmacokinetic parameters in rats treated with famotidine.

## Results

### Dissolution test for famotidine powder mixed with deglutition aid foods

**Figure 1:**
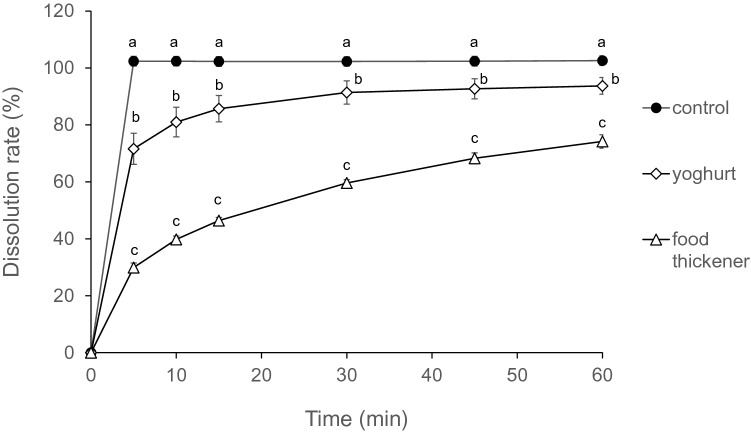
Famotidine powder dissolution rates after mixing with swallowing aid foods. Dissolution rates were measured at 0, 5, 10, 15, 30, 45, and 60 min. Control: only famotidine powder; Yoghurt: famotidine powder mixed with yoghurt; Food thickener: famotidine powder mixed with food thickener. Data are expressed as means ± SD for five samples. Different letters at each time indicate statistically significant differences, determined using one-way ANOVA and Tukey’s post-hoc test.

Famotidine dissolution rate without swallowing aid foods reached 100% after 5 min (Fig. 1). The dissolution rates of famotidine mixed with yoghurt and food thickener were significantly lower than those without swallowing aids at all time points. The dissolution rates of famotidine mixed with yoghurt were significantly higher than those mixed with food thickeners at all time points. The Japanese Pharmacopoeia states that the dissolution rate of 10% famotidine powder should be at least 85% after 15 min^20^. The dissolution rates of famotidine without deglutition aid foods and with yoghurt or food thickener after 15 min were 102.3 ± 1.7, 85.7 ± 4.6, and 46.4 ± 1.1%, respectively.

### Pharmacokinetics of famotidine mixed with deglutition foods in rats

**Figure 2:**
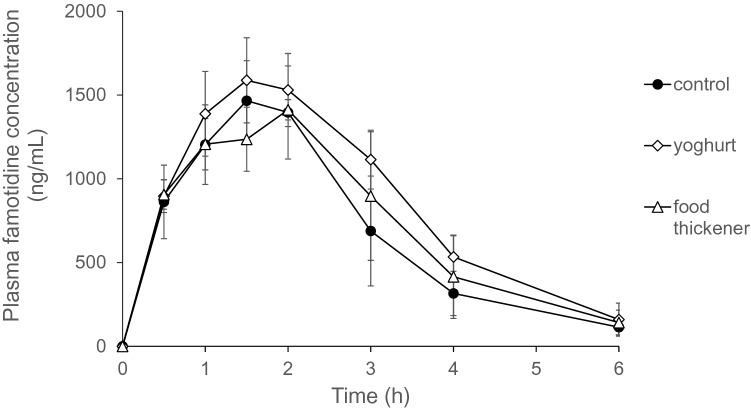
Plasma famotidine levels in rats orally administrated famotidine powder mixed with swallowing aids. Plasma famotidine levels were measured at 0.5, 1, 1.5, 2, 3, 4, and 6 h. Control: rats administered famotidine powder with water; Yoghurt: rats administered famotidine powder mixed with yoghurt; Food thickener: rats administered famotidine mixed with food thickener. Data are expressed as means ± SD for five rats. There was no significant difference between groups in each time point using one-way ANOVA.

Famotidine powder mixed with swallowing aids was orally administered to rats, and plasma famotidine levels were measured up to 6 h later (Fig. 2). There were no significant differences between the plasma famotidine concentrations of the three test groups at any time point. Next, pharmacokinetic parameters were calculated using plasma famotidine concentrations and sampling times (Table 1). The T_max_ values of famotidine without swallowing aid foods, with yoghurt, and with food thickener were 1.7 ± 0.3, 1.4 ± 0.2, and 1.9 ± 0.2 h, respectively. The T_max_ of famotidine mixed with food thickener was significantly lower than that with yoghurt, while there were no significant differences between the control and the other groups. In addition, there were no significant differences among the other parameters in the three groups.

**Table 1:** Pharmacokinetic parameters obtained following oral administration of famotidine powder mixed with swallowing aid foods in rats.

Parameters	Control	Yoghurt	Food thickener
C_max_ (ng/mL)	1488 ± 223	1604 ± 234	1414 ± 64
T_max_ (h)	1.7 ± 0.3^ab^	1.4 ± 0.2^b^	1.9 ± 0.2^a^
AUC_0–6 h_ (ng/mL h)	4089 ± 1029	5158 ± 709	4394 ± 765
t_1/2_ (h)	1.1 ± 0.2	1.2 ± 0.3	1.2 ± 0.2

The experimental designs are the same as those in Fig. 2. Data are expressed as means ± SD for five rats. Different letters in each parameter indicate statistically significant differences, as determined using one-way ANOVA and Tukey’s post-hoc test.

## Discussion

Deglutition aid foods are used to improve medication adherence in patients with dysphagia, but they can interfere with pharmacokinetics^9–13^. Such foods must therefore be carefully selected based on their effect on pharmacokinetics. More options for deglutition-aiding foods appropriate for each drug would be available if yoghurt was used with oral medications. In addition, medication adherence is expected to improve in patients who usually consume yoghurt. In this study, we aimed to assess the applicability of yoghurt with *L. cremoris* FC as a food deglutition aid for famotidine powder. First, we performed a dissolution test using famotidine powder mixed with yoghurt and food thickener. The suppressive effect of yoghurt with *L. cremoris* FC on the dissolution ratio of famotidine was lower than that of the food thickener. The dissolution rate criterion of 10% famotidine powder referred to in the Japanese Pharmacopoeia should be above 85% after 15 min, which is used to guarantee the bioequivalence of generic drugs^20^. The dissolution rate of famotidine with yoghurt met this criterion, whereas that with a food thickener did not. In addition, the FDA published the draft guidance “Use of Liquids and/or Soft Foods as Vehicles for Drug Administration: General Considerations for Selection and In Vitro Methods for Product Quality Assessments” in 2018^21^. This guideline indicates that the dissolution/release testing of drug substances from the dosage form mixed with the food should be carried out in cases where the drug substance is not immediately dissolved in the food. Next, we investigated the pharmacokinetics of famotidine powder containing yoghurt and food thickeners in rats. Compared with the control group, there were no significant differences in any pharmacokinetic parameters between the yoghurt and food thickener groups. Therefore, this result suggests that the administration of famotidine with yoghurt might be a suitable alternative to powder administration as a deglutition aid for patients.

Deglutition foods, especially food thickeners, delayed famotidine powder dissolution in vitro. Yoghurt has a low pH, approximately 4.0–5.0, and is solidified through milk protein aggregation owing to lactic acid fermentation. The isoelectric point of casein, which constitutes 80% of milk protein, is pH 4.6, and the dissolution buffer used in this study was 0.05 M acetic acid/sodium acetate buffer (pH 4.0). Therefore, the drug might have been trapped in the aggregated casein and could not be released. In fact, white grains of milk protein were observed in the test solution from the start to the end of the dissolution test. Meanwhile, food thickeners contain xanthan gum, which is a microbial polysaccharide increasingly utilised for the development and improvement of drug delivery systems owing to its polymeric structure, which delays drug dissolution^22,23^. Thus, a polymeric structure of xanthan gum might have delayed famotidine dissolution in this study.

Although deglutition foods delayed famotidine dissolution in vitro, they did not significantly affect the plasma famotidine concentration in vivo. These results indicated that the data of the dissolution test did not match those of the pharmacokinetics in this study. Famotidine is relatively susceptible to acid-catalysed hydrolysis and is degraded in HCl solutions^24^. Freeks et al.^25^ reported that famotidine inclusion using carboxyl-β-cyclodextrin improves the stability of famotidine under acidic conditions and the bioavailability of famotidine when orally administered to rats. It seems that deglutition foods might inhibit the degradation of famotidine mediated by gastric acid in vivo because of their effect on delaying famotidine dissolution. Consequently, deglutition foods might not have affected famotidine absorption into the blood. In addition, the physical structure of the food is broken through the peristaltic movements of the gastrointestinal tract, which could also be a reason why deglutition foods did not affect plasma famotidine levels in vivo. Moreover, there was a significant difference between the T_max_ values of the food thickener and yoghurt groups, but not between the control and the other groups. This result might indicate that the food thickener delays the T_max_ of famotidine powder and/or that yoghurt expedites it. Therefore, drugs with a designed duration of effect, such as fast- or slow-release drugs, must be used with care.

Polysaccharides can interfere with drug dissolution and are included in not only food thickeners but also yoghurt made with *L. cremoris* FC. When used as a food thickener, xanthan gum is used at 0.05–0.2%^26^. Yoghurt fermented with *L. cremoris* FC contains 30 mg/L (0.003%) exopolysaccharide as a metabolite, hence the amount of polysaccharide in yoghurt is much lower than the amount of xanthan gum used for food thickening^27^. Higher xanthan gum concentration delays drug dissolution^28,29^. Therefore, yoghurt prepared with *L. cremoris* FC, which has small amounts of exopolysaccharides, might only marginally delay drug dissolution.

Overall, the results of this study suggest that yoghurt prepared with *L. cremoris* FC does not remarkably affect the dissolution and pharmacokinetic profiles of famotidine powder. Thus, the administration of famotidine with yoghurt could be a suitable alternative to powder administration as a deglutition aid for patients.

## Methods

### Materials

Famotidine powder under the tradename ‘Gaster® 10%’ was purchased from LTL Pharma Co., Ltd. (Tokyo, Japan). Yoghurt made with *L. cremoris* FC is commercially sold as ‘Caspian Sea Yogurt’ and was provided by Fujicco Co., Ltd. (Kobe, Japan). Yoghurt was stored at 10 °C until use. Xanthan gum-based food thickener under the tradename ‘Tsururinko Quickly’ was purchased from Clinico Co. Ltd. (Tokyo, Japan). To prepare for the experiments, 3 g of food thickener were mixed with 100 mL of soft water, and the mixture was left for 2 min at 25 °C. To equalise the temperature of deglutition aid food for the experiment and for eating, yoghurt and food thickeners were used at 10 and 25 °C, respectively.

### Dissolution test for famotidine powder mixed with deglutition aid foods

The dissolution test was performed using the paddle method according to the Japanese Pharmacopoeia, 18th Edition, at Sunplanet Co., Ltd. (Tokyo, Japan)^20^. Briefly, 100 mg of famotidine powder (equivalent to 10 mg of famotidine) were mixed with 1 g of yoghurt and food thickener, and the mixture was added to 900 mL of 0.05 M acetic acid/sodium acetate buffer (pH 4.0) at 37 ± 0.5 °C. The test solutions were stirred using a paddle at 50 rpm, and 20 mL of samples were collected after 5, 10, 15, 30, 45, and 60 min. The resultant samples were filtered through a 0.45-μm pore filter. In addition, a dissolution test using only famotidine was performed to obtain control data. Sample absorbance was analysed using a UV-1900i spectrophotometer (Shimadzu, Kyoto, Japan) at a wavelength of 266 nm. The famotidine concentration in the samples was calculated using a calibration curve prepared using the absorbance of the famotidine reagent (for biochemistry, > 98%, Fujifilm Wako Pure Chemicals, Osaka, Japan).

### Animals

Animal experiments were performed at Hamaguchi Lab Plus Inc. (Osaka, Japan). All animal experimental procedures were approved by Ethics Committee of Hamaguchi Lab Plus Inc. (approval number: E-21-11-03), based on "Guidelines for proper conduct of animal experiments" by Science Council of Japan. All methods are reported in accordance with ARRIVE guidelines. Eight weeks old male Wistar rats (275–301 g body wight) were supplied by the Jaxon Laboratory Japan (Yokohama, Japan). Rats were kept at 23 ± 2 °C and 55 ± 20% humidity on a 12 h light/dark cycle under conventional conditions and acclimatised for 1 week. All rats were freely allowed access to standard diets, water, and activity. Rats were not sacrificed in this study.

### Oral administration of famotidine powder mixed with deglutition aid in rats

Fifteen rats were randomly divided into three groups of five rats each: control, yoghurt, and food thickener groups. Famotidine powder (100 mg, equivalent to 10 mg famotidine) was mixed with 1 g of yoghurt and food thickener and immediately administered to rats via oral gavage. In the control group, famotidine mixed with water was similarly administered to the rats. Blood samples were collected from the subclavian vein into a heparinied syringe under no anasthesia at 0, 0.5, 1, 1.5, 2, 3, 4, and 6 h. Plasma samples were obtained via centrifugation with lithium heparin and stored at − 80 °C.

### Measurements of plasma famotidine concentration and calculation of pharmacokinetic parameters

Plasma famotidine concentrations were measured using LC/MS/MS at the Sumika Chemical Analysis Service, Ltd. (Osaka, Japan). The pharmacokinetic parameters were analysed using a non-compartment model^30^. C_max_ indicated the peak plasma concentration, and T_max_ indicated the time required to reach C_max_. The C_max_ and T_max_ values were used as the actual measured values. The areas under the curve from 0 to 6 h (AUC_0–6 h_) were calculated using the linear trapezoidal rule for individual concentrations. The elimination rate constant (K_el_) was determined using the linear least squares method with a linear slope during the elimination phase. The half-life (t_1/2_) was calculated as ln2/K_el_.

### Statistical analysis

All results are presented as means ± SD. Differences among the groups were tested using the one-way analysis of variance (ANOVA) with Tukey’s test. *p* < 0.05 was consider statistically significant.

### Ethics approval

Animal experiments were performed at Hamaguchi Lab Plus Inc. (Osaka, Japan) and were approved by its Ethics Committee (permission no. E-21-11-03).

## Data Availability

The datasets generated during the current study are available from the corresponding author on reasonable request.
